# Integrated analysis of lncRNA and mRNA for the apoptosis of porcine ovarian granulosa cells after polyphenol resveratrol treatment

**DOI:** 10.3389/fvets.2022.1065001

**Published:** 2023-01-10

**Authors:** Huibin Zhang, Yangguang Liu, Zheng Han, Qilong Xu, Nannan Zhang, Jinglin Wang, Xianrui Zheng, Yueyun Ding, Zongjun Yin, Xiaodong Zhang

**Affiliations:** ^1^College of Animal Science and Technology, Anhui Agricultural University, Hefei, China; ^2^Anhui Province Key Laboratory of Local Livestock and Poultry, Genetical Resource Conservation and Breeding, Hefei, China

**Keywords:** lncRNA, resveratrol, apoptosis, ovary, porcine

## Abstract

Resveratrol (RES) is a non-flavonoid polyphenol compound that can be involved in follicular development and ovulation. However, the mechanism by which resveratrol regulates the apoptosis of porcine ovarian granulosa cells (POGCs) through long non-coding RNA (lncRNA) is poorly understood. We generated POGCs models of different doses of RES (0, 25, 50, 75, and 100 μM). It was observed that the cell viability was the highest in the 50 μM group, and the highest apoptosis rates were recorded in the 100 μM group. Therefore, a control group (*n* = 3, 0 μM RES group), a low RES group (*n* = 3, 50 μM RES group), and a high RES group (*n* = 3, 100 μM RES group) of POGCs were created for next RNA sequencing. Gene Ontology (GO) indicated that differentially expressed lncRNAs associated with apoptotic process were highly enriched. Kyoto Encyclopedia of Genes and Genomes (KEGG) enrichment analysis of lncRNA target genes found that the Wnt signaling pathway and PI3K-Akt signaling pathway were both enriched. Furthermore, we constructed lncRNA-mRNA networks related to Metabolic and Cell Apoptosis, respectively. In the networks, five key-lncRNAs were screened, which may play a significant role in the process of POGCs metabolism and apoptosis. Furthermore, we focused on the function of a lnc-GAM (lncRNA associated with Granulosa cells Apoptosis and Metabolism) and verified that lnc-GAM could influence cell apoptosis in POGCs development by affecting the mRNA expression of apoptosis-related markers, and also affects the secretion of steroid hormones and related genes expression in POGCs cultured *in vitro*. Our study provides seminal data and important new insights into the regulation of reproductive mechanisms in porcine and other female mammals.

## 1. Introduction

Ovaries play significant roles in mammals, and follicular ovulation, in particular, has a significant impact on mammalian fertility (1). However, more than 99% of porcine follicles develop atresia before ovulation, and only a minority of follicles in the ovary reach the preovulatory stage (2). Proliferation and apoptosis of ovarian granulosa cells play an essential role in the recruitment, selection, ovulation, and atresia of follicles (3). Therefore, elucidating the molecular mechanisms of follicular atresia and granulosa cell apoptosis is of great significance for understanding follicular development and improving the ovulation rate. This will provide a theoretical basis for improving the effective reproduction of sows.

With the popularization and advances in high-throughput sequencing technology in recent decades, a class of substances called long non-coding RNA (lncRNA) has attracted increasing attention and their functions have also been excavated. LncRNAs, a novel type of transcript, have more than 200 nucleotides, do not encode proteins, and are located in the nucleus or cytoplasm. Studies have shown that lncRNAs can affect cell epigenetic regulation, cell cycle progression, and cell differentiation processes, among others (4, 5). At the post-transcriptional level, lncRNAs can target binding microRNA (miRNA), downregulating miRNA expression to regulate gene expression (6). In reproductive activities, lncRNA plays different roles in regulating follicle development, oocyte maturation, and reproductive diseases (7). Although candidate lncRNA associated with ovarian fertility in pigs has been investigated, the mechanism of lncRNA' effects on sow fertility remain elusive (8, 9).

Resveratrol (3,4,5-trihydroxystilbene, RES), is a natural plant non-flavonoid polyphenol and a well-known antioxidant from several plants such as grapes, peanuts, and blueberries (10). Several previous findings demonstrated that RES has been shown to affect processes related to oxidative stress, and exhibits antioxidant, anti-inflammatory, cell apoptosis, cell cycle progression, and growth-inhibiting activities in cancer cell lines and primary cells (11–16). Studies have shown that RES induces apoptosis by inducing the expression of pro-apoptotic proteins and inhibiting the expression of antiapoptotic proteins (17). In addition, RES can also play a role in mammalian reproduction. For example, RES can ameliorate ovarian failure and recovers estrus cycles in polycystic ovary syndrome rats (18). RES 2-hydroxy analogs are biologically active in porcine ovarian granulosa cells (POGCs), where cell viability is almost dose-dependently inhibited by them, and progesterone and estradiol production are affected in porcine ovarian granulosa cells (19). In recent years, there are a substantial number of literature about RES, including a few studies that have involved the differential effects of polyphenols such as RES on lncRNA expression. Interestingly, a study established that RES alleviates skeletal muscle insulin resistance by downregulating lncRNA (20), and inhibits cell apoptosis by suppressing lncRNA in rats' lungs (21). However, the effects of different RES concentrations on apoptosis-related lncRNA and mRNA functions in POGCs have not been elucidated. Therefore, an in-depth study of the effects of different RES concentrations on the proliferation and apoptosis of POGCs is of great significance for understanding the interaction between RES and follicular atresia.

In the study, we hypothesized that RES-induced apoptosis of POGCs may be correlated with different lncRNAs and mRNAs expression in POGCs. Therefore, we used RNA sequencing (RNA-seq) to investigate differentially expressed lncRNAs and mRNAs in POGCs groups with different RES doses, a control RES (0 μM), a low RES (50 μM), and a high RES (100 μM). Meanwhile, GO enrichment and the KEGG pathway were used to clarify the effect of lncRNAs' target genes on cell apoptosis. Some potentially functional lncRNA-mRNA networks have also been revealed. More importantly, we discovered a novel lncRNA associated with granulosa cells apoptosis and metabolism, which we named lnc-GAM. More importantly, the overexpression of lnc-GAM in POGCs can significantly affect cell apoptosis, steroid hormone secretion, and steroid hormone- and apoptosis-related gene expression. These screened data provided references for the apoptosis of POGCs and the application of RES in animal husbandry production. Additionally, this study may also provide the basis for identifying new therapeutic strategies for reproductive diseases, such as PCOS, which causes ovulation failure.

## 2. Materials and methods

### 2.1. Ethics approval and consent to participate

All experiments were performed in accordance with the relevant guidelines and regulations and adhered to the ARRIVE guidelines for reporting animal experiments. This study was conducted according to the principles of the Basel Declaration and recommendations of the Guide for the Care and Use of Laboratory Animals. The protocol was approved by the Ethics Committee of the Anhui Agricultural University under permit no. AHAU20201025.

### 2.2. The POGCs model of RES treatment

Fresh porcine ovaries (from Landrace, ~1 year old) were collected at local abattoir (Hefei, Anhui, China) for the experiments. The ovaries were collected immediately after slaughter, the fatty tissue around the ovaries was removed, and the ovaries were stored in sterile saline solution at 37°C containing 1% penicillin/streptomycin mixture, and transported to the laboratory in a thermos flask within 2 h. After washing the ovaries with phosphate-buffered saline (PBS) at 37°C, healthy small follicles with a pink and well-vascularized follicular wall between 3 and 5 mm in diameter were punctured with a disposable syringe; clear follicular fluid was collected from a number of ovaries (ovaries number >50) to negate any individual animal effects. The follicle suspensions were pooled, centrifuged at 1,000 r/min for 5 min, resuspended and centrifuged again, and the POGCs were harvested immediately.

Briefly, the POGCs were cultured (1 × 10^6^ viable cells/well in 6-wells and 1 × 10^4^ viable cells/well in 96-wells) in a humidified atmosphere (5% CO_2_, 95% air, and 37°C) in the Dulbecco's Modified Eagle Medium (DMEM) medium (Gibco, USA) containing 10% fetal bovine serum (FBS) (Gibco, USA) with 1% penicillin/streptomycin mixture (Gibco, USA) until the POGCs confluence reached up to 80%. Then the medium was removed to establish a model of POGCs treated with different concentrations of resveratrol, then, the treatments received various solutions of RES and were cultured for 24 and 48 h, as followed: 0, 25, 50, 75, and 100 μM. For each treatment, POGCs were allocated to five RES-treated groups with different concentration, each with three replicates. The media and POGCs were collected for subsequent measurements after the 24 and 48 h treatment period.

### 2.3. Methyl thiazolyl tetrazolium assay and cell apoptosis test

Methyl thiazolyl tetrazolium (MTT) assay was performed using an MTT cell proliferation kit (Bestbio, Shanghai, China) according to the manufacturer's instructions. Briefly, POGCs were seeded in 96-well plates, treated with resveratrol (10, 25, 50, 75, and 100 μM), and control were harvested for 24 and 48 h. Absorbance was measured using an Infinite M1000 PRO Microplate Reader (Tecan, Swiss) by optical density at a wavelength of 570 nm. All experiments were repeated at least three times.

POGCs apoptosis was measured by Annexin V-EGFP Apoptosis Detection Kit (Bestbio, Shanghai, China) *via* flow cytometry. The procedure was performed as follows: 1 × l0^6^ cells were collected and washed twice with cold PBS, then resuspended in 500 μL of binding buffer containing 5 μL of propidium iodide (PI) and 5 μL of Annexin V-FITC at 4°C for 30 min. The mixture was incubated for 10 min in the dark at room temperature and then measured by flow cytometry. The FITC and PI fluorescence positive cells were considered apoptotic. Data were analyzed using the FlowJo v7.6 software (Stanford University, CA). All experiments were repeated at least three times.

### 2.4. RNA sample detection, library construction and sequencing

POGCs subjected to control (*n* = 3, 0 μM RES groups), low groups (*n* = 3, 50 μM RES groups), and high groups (*n* = 3, 100 μM RES groups) were used for RNA-seq. Total RNA was isolated and purified from each sample using TRIzol reagent (Invitrogen, Carlsbad, CA, USA) according to the manufacturer's protocol. Then, the possible contamination and degradation of the RNA sample were detected with 1% agarose gel electrophoresis. RNA purity and concentration were examined using the NanoPhotometer^®^ spectrometer (IMPLEN, CA, USA). RNA integrity and quantity were finally measured using the RNA Nano 6,000 assay kit of the Bioanalyzer 2,100 system (Agilent Technologies, CA, USA). RNA with an OD260/D280 absorbance of between 1.8 and 2.0 and RNA integrity number (RIN) equal to or higher than 7.0 was RNA used for further experiments.

For lncRNA sequencing, ~3 μg of RNA of each RNA sample was used for library preparations. Total RNA removed ribosomal RNA (rRNA) to construct chain-specific libraries. Briefly, the ribosomal RNA was depleted from total RNA by the rRNA Removal Kit following the manufacturer's instruction, then, the rRNA-depleted RNA was used to generate sequencing libraries using the EBNext^®^ Ultra™ Directional RNA Library Prep Kit for Illumina^®^ (NEB, USA) following the manufacturer's recommendations. Sequencing was carried out using a 2 × 150 paired-end (PE) configuration on Illumina HiSeq 4,000 platform (Illumina, San Diego, CA, USA).

### 2.5. Sequence map and transcriptome assembly

Clean reads were filtered from raw reads, which were first processed using in-house Perl scripts. In the step, raw 150 bp paired-end reads were filtered by removing reads that were adaptor-containing, low-quality, and ploy N-containing. Clean reads for each sample were aligned to the swine reference genome sus scrofa(pig)-11.1 using the software HISTA. The transcriptome reads alignment results were transferred to the program StringTie for transcript assembly. For miRNA data, raw reads of FASTQ format were first containing poly-N (with 5' adapter contaminants and ploy A or T or G or C, without 3' adapters or the insert tag) and low-quality reads from raw data. The Q20, Q30, and GC content of the clean data were calculated for all RNAs, then, chose the 18- to 35-nt fragments from clean reads to do all the downstream analysis.

### 2.6. lncRNA identification and target gene prediction

All the transcripts were merged using Cuffmerge software, and the unknown transcripts were screened as putative lncRNAs or protein-coding RNAs according to length ≥200 bp, exon number ≥2, and FPKM ≥0.5. Removal of the transcripts with protein-coding capability using CNCI, Pfam, and CPC2 databases.

The transcripts were aligned with lncRNA databases (lncRNAdb and NONCODE) to identify known lncRNAs, novel lncRNAs were named following rules of HGNC (The HUGO Gene Nomenclature Committee) and the characteristics of novel lncRNA were compared with known lncRNA and mRNA. The co-expression relationship between lncRNA and mRNA was described by Trans. The basic principle of the transaction is that the function of lncRNA is not related to the location of the coding gene, but to the protein-coding genes that they co-expressed. According to the expression correlation coefficient cor was > 0.9 between lncRNA and mRNA.

### 2.7. RNA quantification and functional enrichment analysis

The expression level of mRNAs and lncRNAs were quantified based on fragments per kilobase of transcript per million fragments mapped (FPKM) using the StringTie software, the edgeR software package was used to detect differentially expressed lncRNAs (DELs) with criteria of log_2_|fold change|≥1 and *P*-adjust value < 0.05, differentially expressed mRNAs (DEMs) were similarly screened out.

Gene Ontology (GO) enrichment analysis was performed on the target gene candidates of the DELs by GOseq (22). Pathway analysis for genes was provided, based on the KEGG (Kyoto Encyclopedia of Genes and Genomes; http://www.genome.jp/kegg) database. KOBAS software was used to test the statistical significance of the target gene candidates of the DELs enrichment in KEGG pathways.

### 2.8. Validation of lncRNA and mRNA expression by qRT-PCR

To test the accuracy of the sequencing results, we selected four DELs and DEMs for RT-qPCR verification. We used a cDNA synthesis kit to reverse transcribe the extracted total RNA into cDNA. Primers of mRNAs and lncRNAs were designed and synthesized by TsingkeBio (Beijing, China). Supplementary Table 1 lists the primers used in this study. Real-time quantitative PCR reactions were performed on a Bio-Rad CFX96 Real-Time Detection System (BioRad, CA, United States) using an iTaq Universal SYBR Green Supermix Kit and ran each sample in triplicate to ensure the accuracy of the quantitative results. The 2^−Δ*ΔCt*^ method was used to calculate the relative expression of the target genes, GAPDH and β*-actin* were used as internal controls for mRNAs and lncRNAs, respectively.

### 2.9. Plasmid construction, cell transfection, cell proliferation assay

To construct lnc-GAM overexpression plasmids, the full lengths of lncR-GAM gene were amplified by PCR using Tks GflexTM DNA polymerase (Takara, Japan) and cloned into pcDNA3.1 vector. And the recombinant vectors were named pcDNA3.1-lncGAM and pcDNA3.1-NC. Transfection of POGCs with plasmids was conducted with Lipofectamine 3,000 reagent (Invitrogen, USA) in accordance with the manufacturer's protocol.

After plasmids transfection, cell viability was determined using the CCK-8 assay according to the manufacturer's protocol and measured at 450 nm. Also, EdU Cell Proliferation Assay Kit (Bestbio, Shanghai, China) was used following the manual book. Cell proliferation was analyzed under a fluorescent microscope.

### 2.10. E2 and P4 detection

ELISA was used to detect the levels of estrogen (E2) and progesterone (P4) in GCs with different treatments according to the protocol of the E2 ELISA Kit and the PROG ELISA Kit (Enzyme-linked Biotechnology Co., Ltd., Shanghai, China). Briefly, the POGCs were treated for 48 h in 6-well plates, and 1 mL supernatants were collected from the POGCs cultures. Then, the Standards were diluted serially and test samples were incubated in an ELISA plate. Finally, the absorbance at 450 nm was determined by using a Fluorescence/Multi-Detection Microplate Reader (Bio Rad, USA).

### 2.11. Statistical data analysis

Data are presented as means ± standard deviation (SD) for at least triplicates. The GraphPad Prism (version 5.0) software (San, Diego, CA, United States) was used to analyze the results of RT-qPCR and for graphing.

Differences between different groups were analyzed by a two-tailed Student's *t*-test. A *p*-value < 0.05 was considered to be statistically significant. The significance was marked as ^*^P < 0.05 and ^**^P < 0.01.

**Figure 1:**
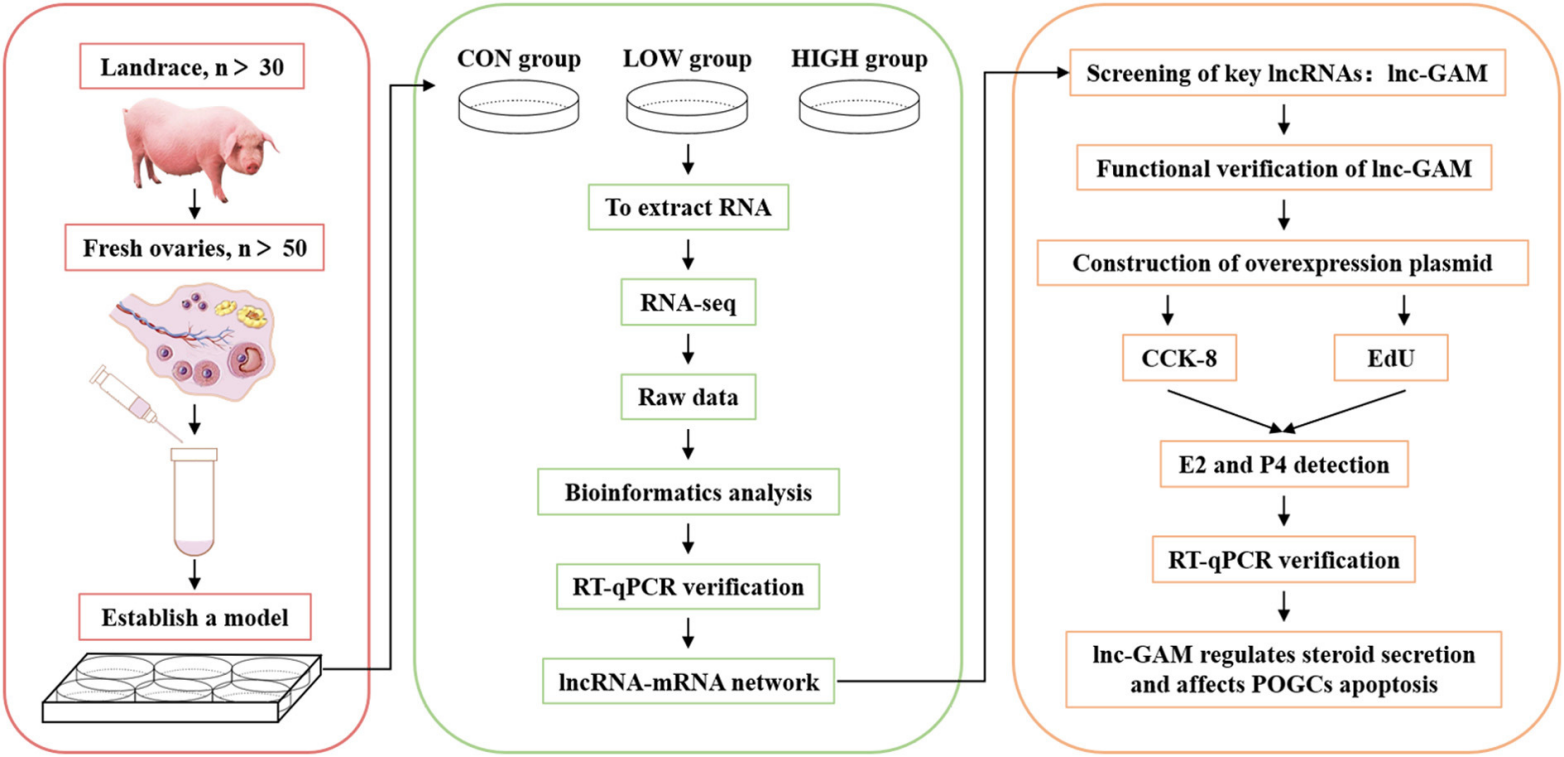
Workflow chart of sequencing data processing and verification.

For the above experiments, we carried out a workflow flow chart (Figure 1).

## 3. Results

### 3.1. Model of resveratrol treatment in POGCs

**Figure 2:**
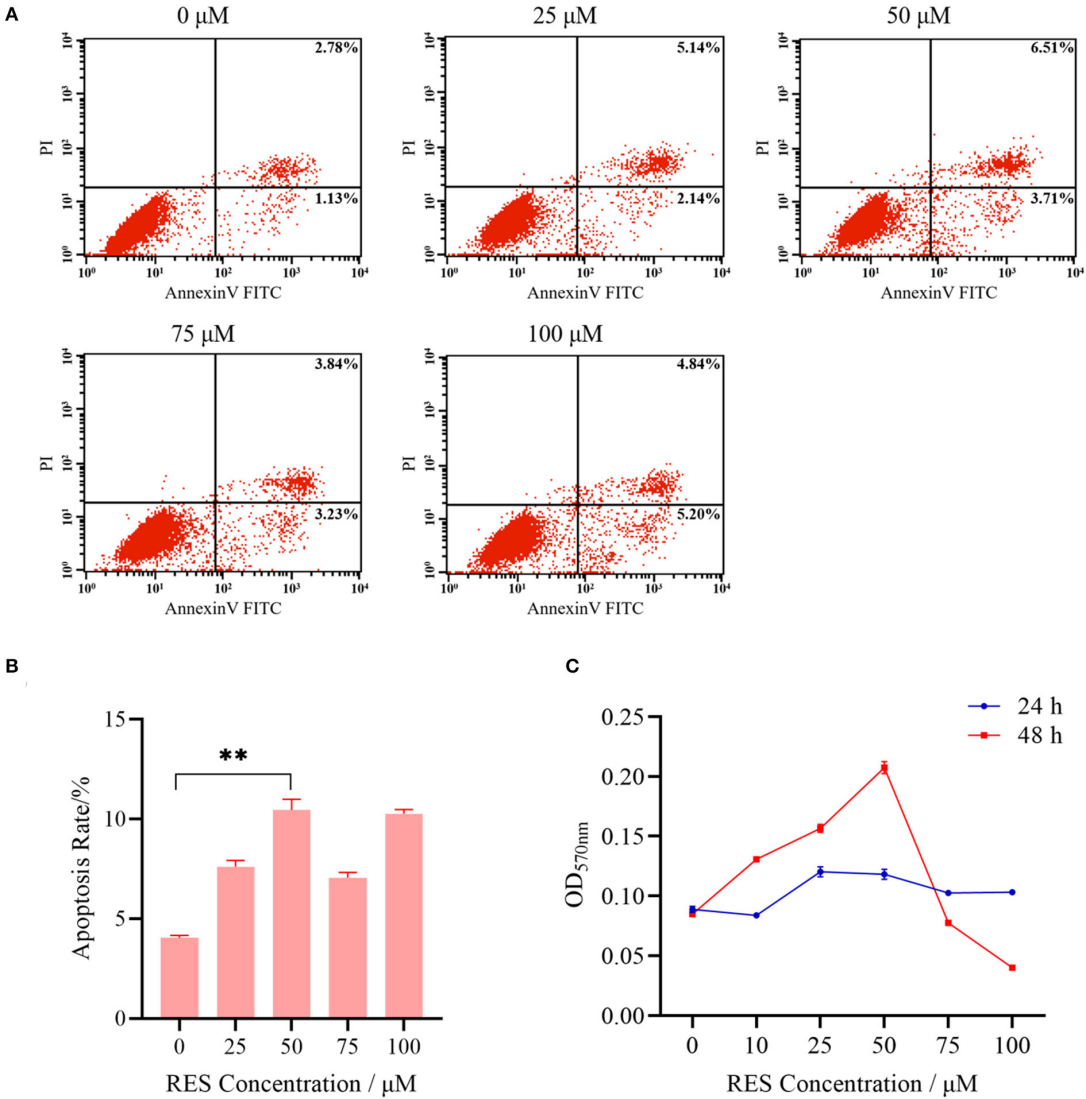
Basic characteristics of POGCs models in different RES (0, 25, 50, 75, and 100 μmol/L) groups. **(A)** Analysis of apoptosis level of POGCs receiving differing solutions of RES followed by Annexin V-FITC/PI kit and flow cytometry. **(B)** Apoptosis rate of POGCs in different RES concentration groups. **(C)** MTT assay was performed to assess the effect of RES on POGCs proliferation. ^**^P < 0.01.

The flow cytometry and MTT assay were first applied to determine the POGCs apoptosis and proliferation in resveratrol (RES) concentration groups (0, 10, 25, 50, 75, and 100 μM). Results showed that the lowest POGCs apoptosis occurred in 0 μM group, and the highest rate of apoptosis was observed in 100 μM group (Figures 2A, B), followed by 50 μM group. And the proliferation rate of POGCs gradually increased with increasing RES concentration at 48 h (Figure 2C). The highest proliferation rate was achieved when the concentration reached 50 μM. However, the apoptosis rate of POGCs is increasing all the time compared with 0 μM group. These results suggest that RES regulates POGCs differentiation and apoptosis. Therefore, we selected these three groups of POGCs for RNA-seq: control (RES concentration = 0 μM), low group (RES concentration = 50 μM), and high group (RES concentration = 100 μM).

### 3.2. Summary of lncRNA sequencing results

**Figure 3:**
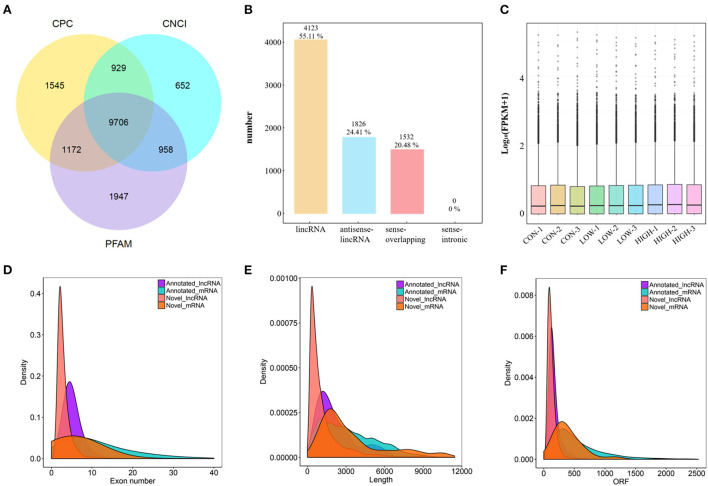
Characterization of lncRNA and mRNA in POGCs. **(A)** Coding potential analysis *via* CNCI (Coding-Non-Coding Index), CPC (Coding Potential Calculator), PFAM (Protein Families database). Those sequences simultaneously shared by the above three tools were selected as candidate lncRNAs. **(B)** The classification of identified lncRNAs. **(C)** Boxplot showing the expression features of lncRNA in POGCs. FPKM, fragments per kilobase million. **(D–F)** Density distribution diagram showing the expression features of exon number **(D)**, length **(E)**, and opening reading frame (ORF) **(F)** of annotated lncRNAs, novel lncRNAs, and mRNAs in POGCs.

After low-quality reads were removed, 78.8–104.7 M paired-end reads (2 × 150 bp in the length) for the mRNAs and lncRNAs, were acquired from the three groups (CON, LOW and HIGH) by Illumina Hiseq 2,500 platform (Supplementary Table 2). Subsequently, we identified 18,172 novel lncRNAs based on analysis by the Coding Potential Calculator (CPC), Coding-Non-Coding Index (CNCI), and Protein Families database (PFAM) (Figure 3A), which included 4,123 lncRNAs (55.11%), 1,826 antisense-lncRNA (24.41%), and 1,532 sense-overlapping (20.48%) (Figure 3B). The expression volume box plots of each sample were constructed based on FPKM. The results showed that the distribution of each box plot was flat (Figure 3C), indicating that the expression symmetry and distribution of lncRNAs in each sample are correct. Meanwhile, although small differences in lncRNA expression levels could be observed in the samples between groups, the distribution of lncRNA was similar. To investigate the characteristic differences between lncRNAs and mRNAs in POGCs, we compared the number of overlapping mRNAs in the LOW, HIGH, and CON groups and found that lncRNAs and mRNAs had essentially the same range of length distributions, but compared to mRNAs, annotated and novel lncRNAs were smaller in size, fewer exons, and fewer open reading frames (Figures 3D–F).

### 3.3. Differential expression and cluster analysis of lncRNAs and mRNAs

**Figure 4:**
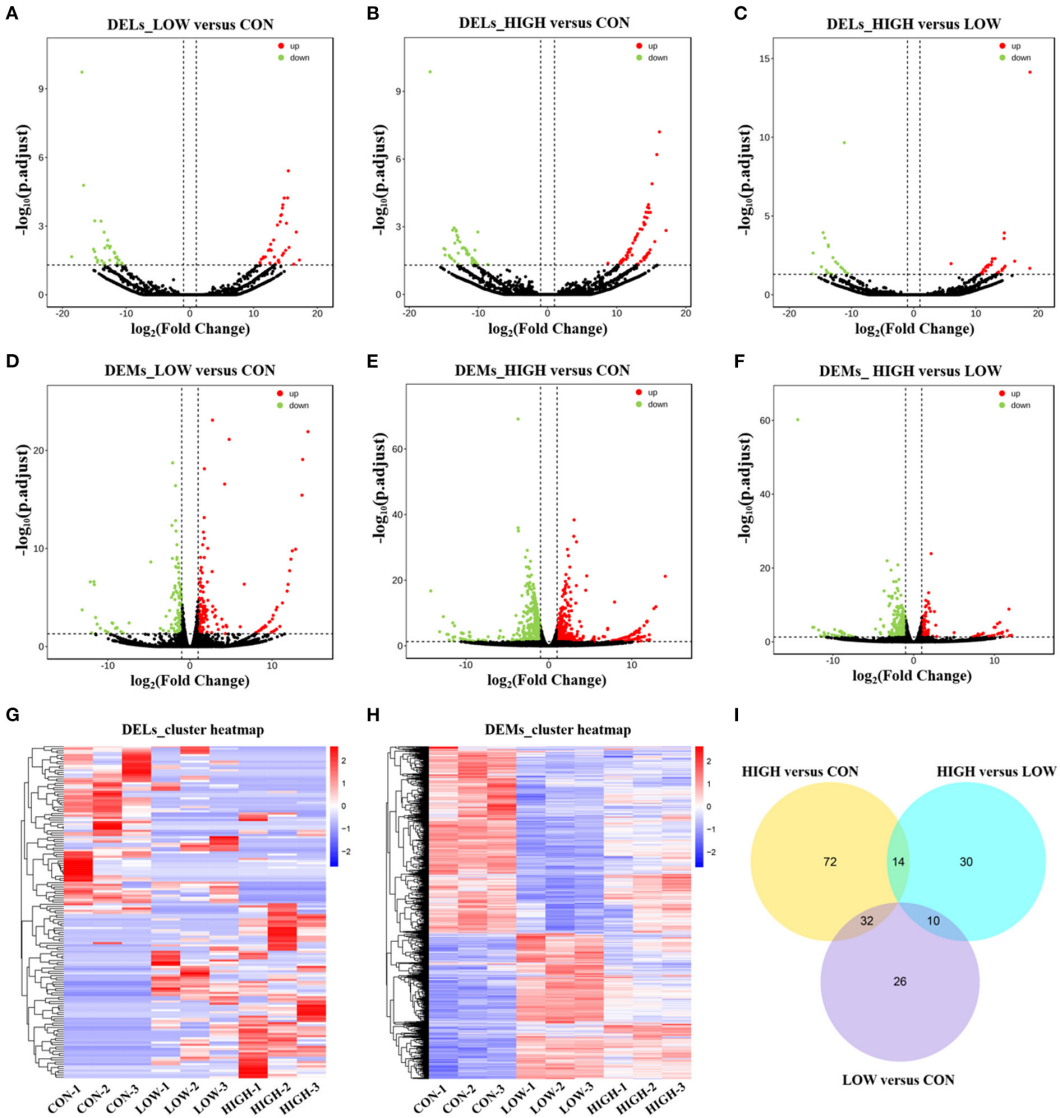
Expression Profiles of lncRNAs and mRNAs in POGCs. **(A–C)** Volcano plot indicating up– and downregulated lncRNAs of POGCs in different RES treatment groups (LOW vs. CON, HIGH vs. CON, HIGH vs. LOW); up– and downregulated genes are colored in red and green, respectively. **(D–F)** Volcano plot indicating up– and downregulated mRNAs of POGCs in different RES treatment groups (LOW vs. CON, HIGH vs. CON, HIGH vs. LOW); up– and downregulated genes are colored in red and green, respectively. **(G, H)** Heatmap of lncRNAs **(G)** and mRNAs **(H)** showing hierarchical clustering of changed lncRNAs and mRNAs of POGCs in different RES treatment groups; up– and downregulated genes are colored in red and blue, respectively. **(I)** Venn diagrams of DELs in response to different treatments.

Considering there were three different treatment concentrations, we performed the analysis in the three comparisons (LOW vs. CON, HIGH vs. CON, and HIGH vs. LOW), respectively. Differentially expressed lncRNAs (DELs) and differentially expressed mRNAs (DEMs) in the samples were shown using volcano plots and heatmaps. And there were 68 (34 upregulated and 34 downregulated), 118 (65 upregulated and 53 downregulated), and 54 (31 upregulated and 23 downregulated) DELs identified in the LOW vs. CON (Figure 4A, Supplementary Table 3A), HIGH vs. CON (Figure 4B, Supplementary Table 3B), and HIGH vs. LOW groups (Figure 4C, Supplementary Table 3C), respectively. In addition, there were 278 (153 upregulated and 125 downregulated), 905 (491 upregulated and 414 downregulated), and 373 (115 upregulated and 258 downregulated) DEMs identified in the LOW vs. CON (Figure 4D, Supplementary Table 4A), HIGH vs. CON (Figure 4E, Supplementary Table 4B), and HIGH vs. LOW groups (Figure 4F, Supplementary Table 4C), respectively. Hierarchical clustering of the DELs (Figure 4G) and DEMs (Figure 4H) revealed the expression patterns of the individuals for the same three comparisons. Moreover, among the three groups, a total of 26, 72 and 30 specific DELs were observed in LOW vs. CON, HIGH vs. CON, and HIGH vs. LOW groups by Venn diagram analyses, respectively (Figure 4I). It is noteworthy that the specific expression of lncRNAs in Venn diagrams, we found lncRNA TCONS_00201518 was highly expressed and significantly upregulated [log2 (Fold Change) = 15.56] in LOW vs. CON group. Meanwhile, lncRNA TCONS_00043772 was significantly upregulated [log2 (Fold Change) = 14.69] in HIGH vs. CON group. The results indicated that these DELs may play important roles in the regulation of POGCs apoptosis induced by RES.

### 3.4. Validation of RNA sequencing using RT-qPCR

**Figure 5:**
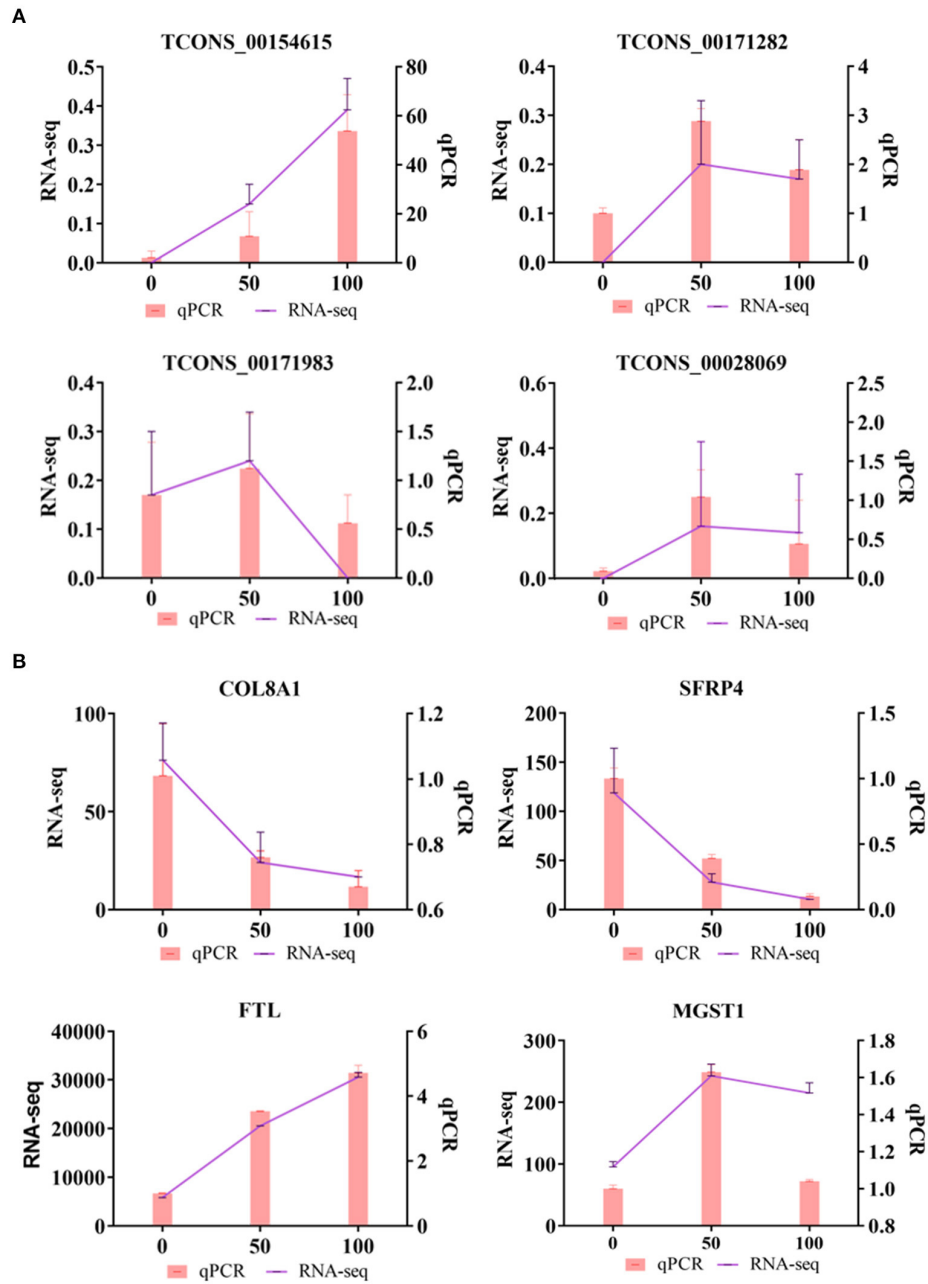
Quantitative real-time PCR (qRT-PCR) validations. **(A)** Verification of DELs by qRT-PCR. **(B)** Verification of differentially DEMs by qRT-PCR.

To verify the sequencing reliability, nine lncRNAs were randomly selected from the three comparison groups and subjected to RT-qPCR testing. The qPCR results showed that the expression levels of TCONS_00154615 were the lowest in CON group and the highest in HIGH group. TCONS_00171282, TCONS_00171983, and TCONS_00028069 were all higher in the LOW group, and the expression levels of these lncRNAs were relatively lower in the CON group and the HIGH group. These results were consistent with our RNA-seq. In addition, the expression levels of COL8A1 and SFRP4 genes in CON, LOW, and HIGH groups decreased in turn, which was consistent with the sequencing results. The mRNA expression levels of FTL and MGST1 were also shown to be consistent in RNA-seq (Figure 5).

### 3.5. Target gene prediction and functional analysis of DELs

**Figure 6:**
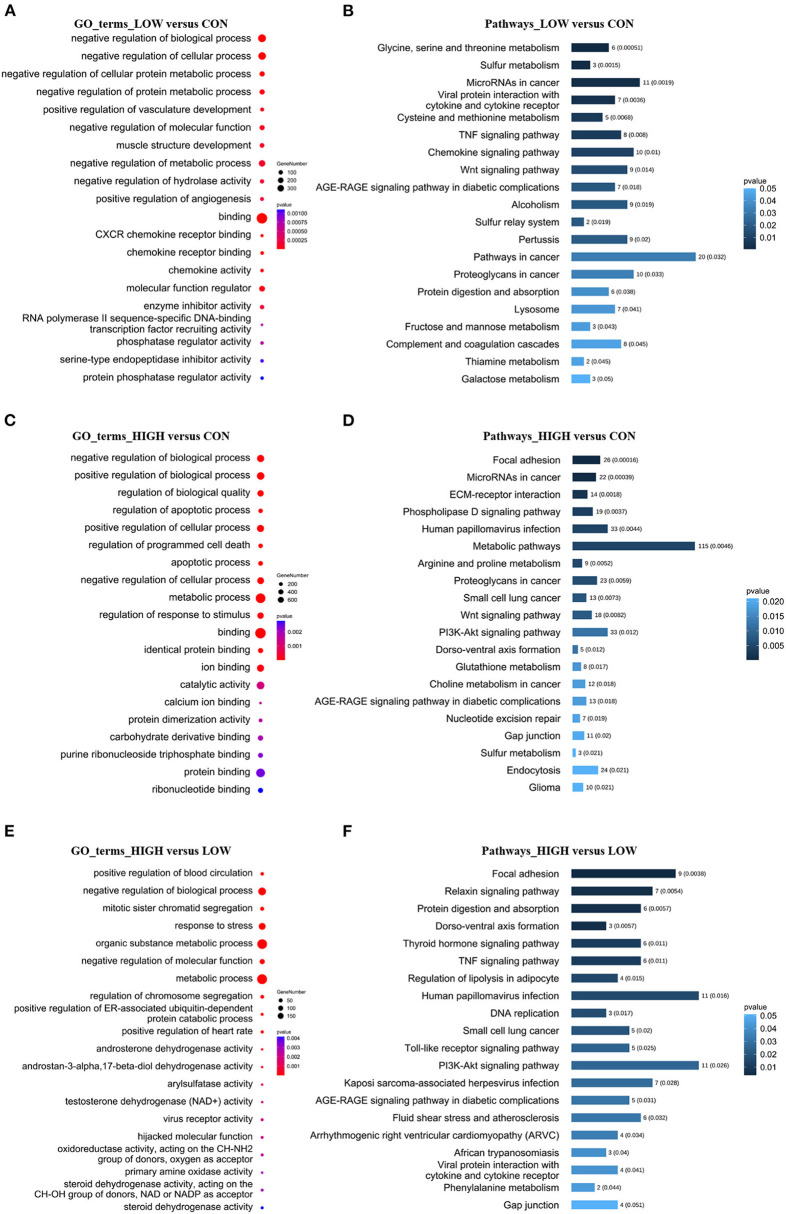
GO and KEGG Analyses of differentially expressed lncRNA target genes in POGCs in different RES-treatment groups. **(A, C, E)** GO categories (biological process, molecular function) of target genes for DELs. **(B, D, F)** KEGG analysis of target genes for DELs. The size and color of each bubble represents the number of genes in each GO terms and *p*-value, respectively.

**Figure 7:**
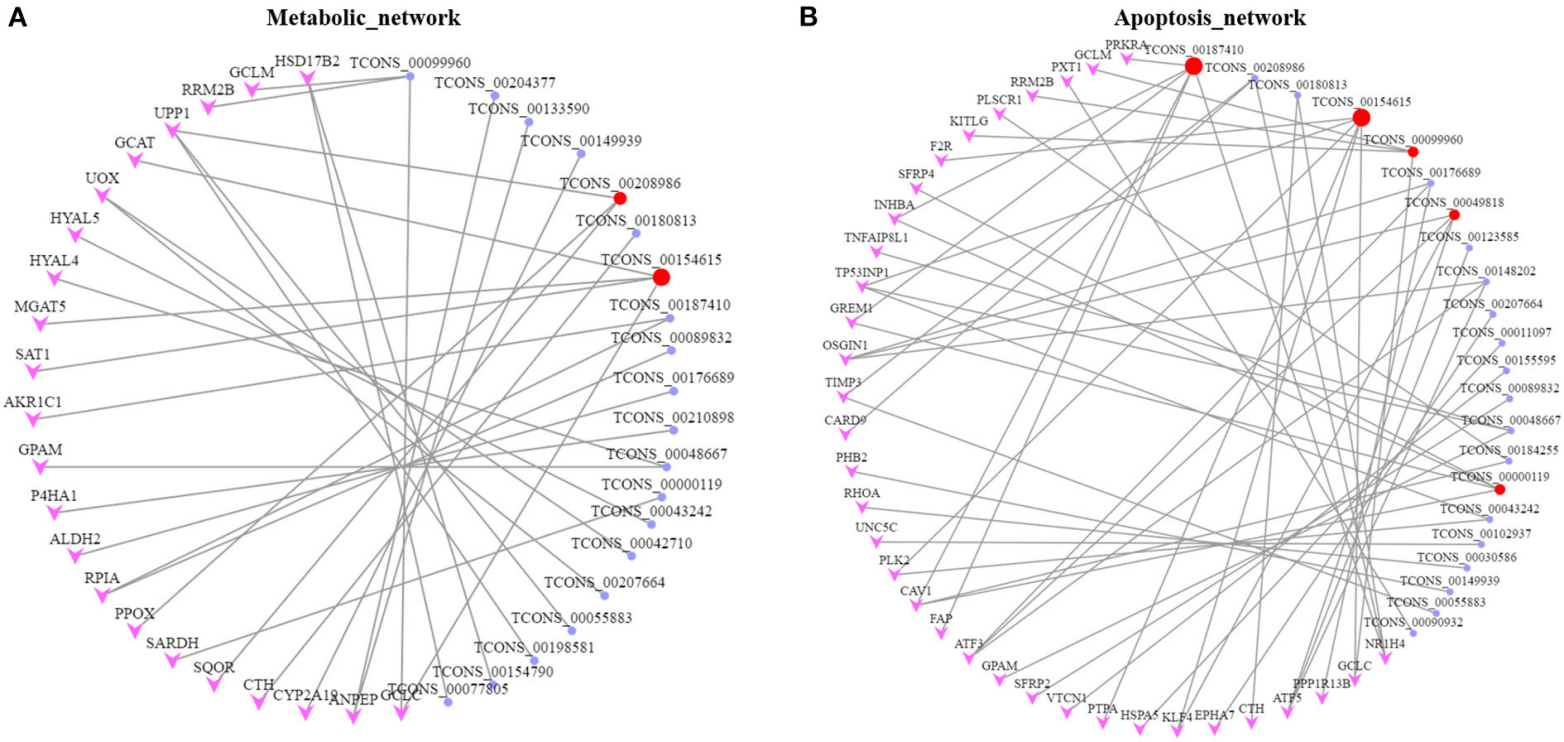
Networks for lncRNA-mRNA interaction. **(A)** lncRNA-mRNA network for Metabolic. **(B)** lncRNA-mRNA network for Apoptosis. The target genes and lncRNAs colored in pink and purple, respectively. Key lncRNAs are colored in red.

To further explore the physiological and molecular functions of DELs on apoptosis in POGCs induced by RES. For 68 DELs in the LOW vs. CON, target gene prediction based on the correlation between lncRNA and mRNA expression, with a Pearson correlation coefficient >0.95 as the threshold (Supplementary Table 5A), we predicted 520 target genes. Moreover, we predicted 1,404 and 362 target genes of DELs in HIGH vs. CON (Supplementary Table 5B) and HIGH vs. LOW groups (Supplementary Table 5C), respectively. Meanwhile, Gene Ontology (GO) annotation and Kyoto Encyclopedia of Genes and Genomes (KEGG) enrichment analysis were conducted using the identified potential target genes of DELs (Figure 6, Supplementary Tables 6, 7). The results of GO enrichment showed that these target genes were involved in the regulation of various biological processes such as negative regulation of biological process, metabolic process, and apoptotic process (Figures 6A, C, E, Supplementary Table 6). Hence, it can be speculated that these DELs may participate in the apoptosis response by regulating their target genes during RES-induced POGCs apoptosis. It is noteworthy that metabolic process was enriched among the three comparison groups, especially in HIGH vs. CON and HIGH vs. LOW groups, suggesting that metabolic process might play a critical role in RES-induced POGCs apoptosis. In KEGG analysis, some interesting signaling pathways are enriched, which are related to cell development, including Wnt signaling pathway, PI3K-Akt signaling pathway, FoxO signaling pathway, and DNA replication signaling pathway (Figures 6B, D, F, Supplementary Table 7). Notably, the Metabolic signaling pathway was enriched in HIGH vs. CON (Figure 6D), this finding is consistent with the GO term enrichment results. Therefore, we constructed a lncRNA-mRNA networks for “Metabolic” based on DELs and DEMs (Figure 7A, Supplementary Table 8A). The 20 lncRNAs involved in this function target 21 genes, of which lncRNA TCONS_00154615 has 4 targeted genes and is identified as a key-lncRNA for this function. Furthermore, we screened the GO terms and KEGG signaling pathways associated with “Cell Apoptosis”, and constructed a lncRNA-mRNA network with 22 lncRNAs involved in this function target 34 genes (Figure 7B, Supplementary Table 8B). In the Apoptosis network, we found lncRNA TCONS_00154615, which is also involved in the Metabolic network. The result shows that lncRNA TCONS_00154615 plays a significant role in the Apoptosis network. In addition, lncRNA TCONS_00187410, lncRNA TCONS_00099960, lncRNA TCONS_00049818, and lncRNA TCONS_00000119 may also play key roles in the apoptosis.

### 3.6. Overexpression of lnc-GAM regulated POGCs proliferation *in vitro*

**Figure 8:**
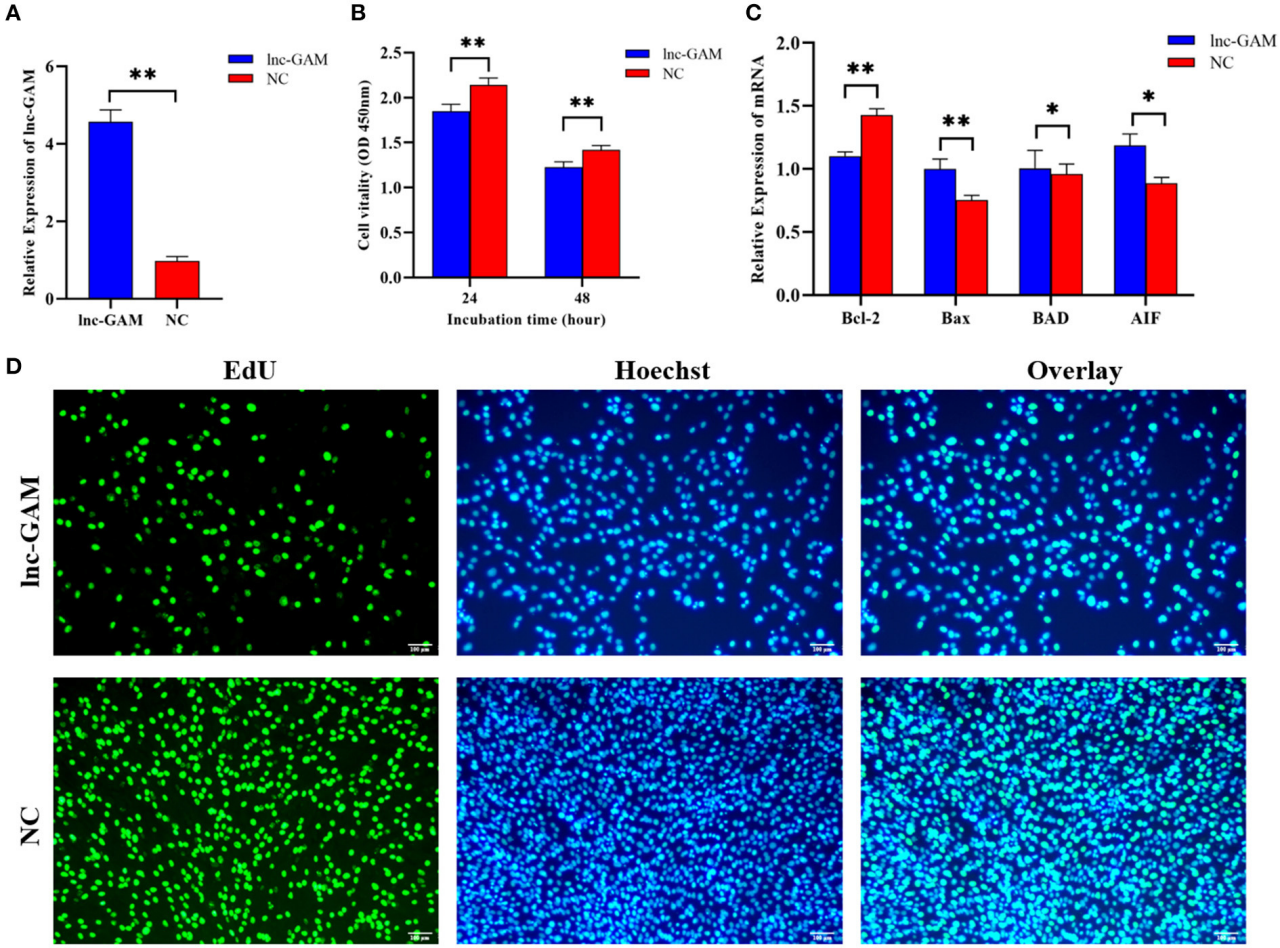
Overexpression of lnc-GAM inhibited POGCs proliferation. **(A)** Graph showing the expression level of lnc-GAM in POGCs after transfection *via* RT-qPCR (*P < 0.05, **P < 0.01, the same below). **(B)** Graph showing the viability of POGCs treated with lnc-GAM was measured by using CCK-8 at the indicated time points. **(C)** The mRNA levels of apoptosis-related factors were estimated by quantitative real-time PCR. GAPDH served as the internal reference. **(D)** Graphs showing the proportion of proliferation cells *via* EdU assay. The number of cells was detected by staining with Hoechst (blue), as the proliferated cells were detected by staining with EdU (green). The result was analyzed by fluorescence microscope.

To investigate the role of lnc-GAM in the POGCs, lnc-GAM overexpression and control vectors were constructed. And the overexpression efficiency of lnc-GAM was verified by RT-qPCR in two groups: a positive control group (pcDNA3.1), an overexpression group (pcDNA3.1-lncGAM) (Figure 8A). We measured cell proliferation after transfection. EdU and CCK8 assays consistently showed that upregulated lnc-GAM inhibited cell proliferation (Figure 8B) and reduced the proportion of proliferating cells in total cells (Figure 8D). These findings suggest that upregulated lnc-GAM may inhibit POGCs proliferation. Furthermore, we found lnc-GAM overexpression significantly promoted the expression of apoptosis-related mRNAs (Bax, BAD and AIF) and significantly inhibited the mRNA expression of the anti-apoptosis-related gene (Bcl-2) (Figure 8C). Taken together, the data suggested that lnc-GAM can inhibited POGCs proliferation through affecting mRNA expression of apoptosis- (Bax, BAD and AIF) related makers.

### 3.7. lnc-GAM regulates steroid secretion by POGCs

**Figure 9:**
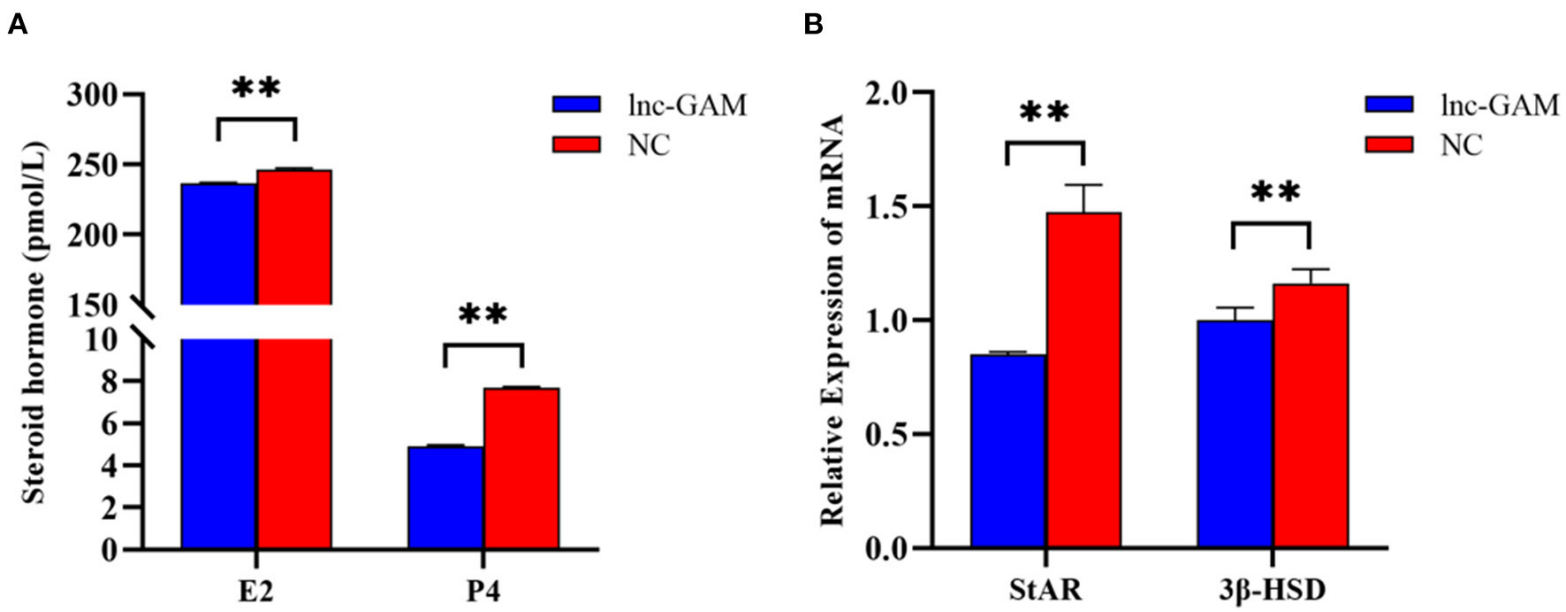
lnc-GAM effects on steroid secretion by POGCs. **(A)** Estradiol and progesterone contents in the supernatant of cells with different lnc-GAM expression profiles. **(B)** The mRNA levels of steroidogenesis-related factor were estimated by quantitative real-time PCR. GAPDH served as the internal reference. The lnc-GAM refers to pcDNA3.1-lncGAM, and NC refers to pcDNA3.1, the same blow. ^**^P < 0.01.

The levels of E2 and P4 in cell-free supernatant were determined by ELISA after transfection of pcDNA 3.1-lncGAM in POGCs for 24 h and 48 h to explore the effect of lnc-GAM on steroid hormone synthesis. lnc-GAM overexpressing cells showed enhanced secretion of E2 and P4 (Figure 9A) compared with pcDNA3.1-NC. In addition, the expression levels of steroidogenesis-related genes were assessed by qRT-PCR, and the results are displayed in Figure 9B. When lnc-GAM was overexpressed, the expression levels of steroidogenic acute regulatory (StAR) protein and 3β-hydroxysteroid dehydrogenase (3βHSD) were significantly increased in POGCs. These results suggested that lnc-GAM affected steroid hormone synthesis and its related-gene expression in POGCs.

## 4. Discussion

The ovary is one of the important reproductive organs of female animals, regulating follicle development and hormone secretion (23). Proliferation and apoptosis are natural physiological processes that occur naturally during the growth and development of ovarian follicles (24). In particular, the apoptosis of POGCs plays a vital role in the induction of atresia in porcine ovarian follicles (25). Therefore, granulosa cells play an important role in follicular atresia, follicle growth, and the maintenance of the estrous cycle (24). In follicular atresia, reproductive hormones, pro- and anti-apoptotic factors may be involved, and many studies have shown that most of these factors contribute to the atresia of follicles by triggering the apoptosis of granulosa cells, such as in chickens, mice, and pigs (26, 27). Different research demonstrated that RES acts as a natural antioxidant in low concentrations in normal cells (28). High-dose RES, instead, may prompt pro-oxidant effects, inducing mitochondrial-dependent cell death (28, 29). In addition, RES can promote apoptosis to reduce luteinization in rat ovaries *in vitro* (30), exerting its effects by interacting with multiple cellular targets and regulating various signaling pathways (31). In our study, similar to previous studies, POGCs models of different doses of RES (0, 25, 50, 75, and 100 μM) were generated. We observed that the highest cell viability was recorded in the 50 μM group and the highest apoptosis rates were recorded in the 100 μM group. The results indicate that RES has important effects on the proliferation and apoptosis of POGCs. However, information regarding the roles of lncRNAs in POGCs apoptosis is limited, and no studies have reported the roles of lncRNAs in the RES-induced apoptosis of POGCs. Thus, a control group (*n* = 3, 0 μM RES group), a low RES group (*n* = 3, 50 μM RES group), and a high RES groups (*n* = 3, 100 μM RES group) of POGCs were created for next RNA sequencing.

Due to the low conserved nature of lncRNA sequences among species, bioinformatic methods are required to screen and identify lncRNAs. The analysis is based on transcript length, the number of exons, and coding potential (32). In our studies, a total of 240 DELs were observed in POGCs treated with RES at 0, 50, and 100 μM. And there were 68, 118, and 54 DELs identified in the LOW vs. CON, HIGH vs. CON, and HIGH vs. LOW groups, respectively. By comparing the existing literature with the results of this experiment (33), differences in the type and expression levels of lncRNAs at different concentrations of induction were observed. Thus, it is likely that lncRNAs are involved in the regulation of apoptosis and metabolism induced by RES. To clarify the potential functions of DELs, DELs target gene prediction and GO functional enrichment analysis revealed that 240 DELs in RES-induced POGCs might regulate the expression of 2,286 target genes belonging to several significant signaling pathways. Some such pathways include the Wnt signaling pathway, PI3K-Akt signaling pathway, FOXO signaling pathway, Apoptosis signaling pathway, and Metabolic signaling pathway, which were confirmed to have participated in the regulation of cell differentiation, apoptosis, and metabolism. Recent studies have shown that the Wnt signaling pathway plays an important role in the development and regulation of ovarian folliculogenesis and ovulation (34). Zhou et al. reported that follicular fluid-derived exosomal miR-18b-5p reduces PTEN expression and promotes the activation of the PI3K/Akt/mTOR signaling pathway to inhibit polycystic ovary syndrome development (35). FOXO3, which is a FOXO family member, and Cui et al. demonstrated that activation of the PI3K-FOXO3 pathway restores ovarian function in ovariectomized rats (36). These existing studies have shown that the above-mentioned signaling pathways we screened are closely related to the occurrence, development, and regulation of apoptosis (35, 36). Furthermore, Basini et al. (19) found that RES-Analog inhibited granulosa cell growth, while it stimulated steroidogenesis. Whenever RES acts as a pro-oxidant molecule *in vitro*, it can enhance cellular metabolism, and activate cellular damage, and apoptotic pathways, which are usually followed by phospho-PKB/Akt downregulation (29). Recently, it was also shown that RES induces caspase-dependent cell death in ovarian cancer cells (37). Interestingly, apoptotic and metabolic pathways were also significantly enriched in our analysis. Based on the above results, it was speculated that these DELs are likely involved in regulating the process of POGCs apoptosis induced by RES *via* their apoptosis- and metabolism-related target genes.

Based on the data presented here, in conjunction with the literature discussed above, it is clear that further examination of lncRNA expression profiles in the RES-induced apoptosis model of POGCs is needed to clarify the molecular mechanisms regulating the apoptotic process. However, few reports have investigated the role of lncRNA in the mechanism of RES-induced cell apoptosis. It was reported that RES inhibited cancer cell survival, while the knockdown of lncRNA H19 resulted in increased sensitivity to RES therapy (38). Liu et al. (20) showed that RES could promote skeletal muscle insulin resistance *via* a competitive endogenous RNA (lncRNA NONMMUT044897.2/miR-7051-5p/SOCS1 pathway). In addition, RES inhibits cell apoptosis by suppressing lncRNA XLOC_014869 in lung injury rats (21). In our study, we focused on apoptotic and metabolic pathways to find key lncRNAs involved in RES-induced apoptosis in POGCs, and as a flavonoid polyphenol, RES may be related to the potential mechanism of POGCs apoptosis induced by organic substance metabolic. Then, we constructed lncRNA-mRNA networks for “Metabolic” and “Apoptosis”, and five novel lncRNAs were identified that may be involved in RES-induced regulation of POGCs metabolism and apoptosis. Among them, the lncRNA TCONS_00154615 was enriched by the “Metabolic Network” and “Apoptosis Network”, and was considered to be the key lncRNA. Thus, we suspected that lncRNA TCONS_00154615 plays an important role in POGCs development and refer to this lncRNA as lnc-GAM for convenience.

Subsequently, we specifically investigated the specific effects of lnc-GAM on POGCs *in vitro*. We found that cell proliferation viability decreased significantly after overexpression of lnc-GAM in POGCs. Hence, the effect of lnc-GAM on gene expressions about the apoptosis of cells was detected. The results were shown lnc-GAM induced Bcl-2 expression, and down-regulated mRNA expression of Bax, BAD, and AIF. Obviously, lncRNA affected the expression of apoptosis-related genes and inhibited cell proliferation. E2 and P4 steroid hormones are well known to regulate the expression of genes involved in ovulation and luteinization. Therefore, this study examined the effect of lnc-GAM on E2 and P4. In our study, we found that lnc-GAM significantly decreased the concentration of E2 and P4 by inducing the expression of steroidogenic enzymes. Lnc-GAM inhibited POGCs steroidogenesis by regulating the expression of 3β-HSD and STAR. Previous studies have pointed out that PITX2 regulates the secretion of E2 and P4 from granulosa cells *via* the Wnt/β-catenin pathway and alters granulosa cells' proliferation and steroidogenesis (39). Liu et al. (40) confirmed that the Chi-miR-439-3p-JAK3 regulatory pathway can affect the steroid hormones E2 and PROG. Our results indicated that the regulation of steroidogenesis processes in porcine ovarian granulosa cells was similar to previous research in other species (39, 40). All results revealed that lnc-GAM has a potential influence on the biological process of POGCs which regulated cell viability, cell proliferation, steroidogenesis, and E2 and P4 synthesis in POGCs.

In brief, high-throughput sequencing results suggest that DELs may play a crucial role in the regulation of RES-induced apoptosis in POGCs by regulating their target genes to participate in metabolism-related and apoptosis-related signaling pathways. Furthermore, we confirmed that lnc-GAM decreased POGC proliferation by cell apoptosis-related genes including Bcl-2, Bax, BAD, and AIF. And lnc-GAM also inhibited the secretion of E2 and P4 in POGCs. And these confirm the significance of the key lncRNA we screened.

## 5. Conclusions

Here, we systematically identified the expression profiles of lncRNAs and mRNAs involved in RES-induced apoptosis in POGCs. Abundant lncRNAs were expressed in RES-induced POGCs. These DELs might be involved in metabolism and apoptosis *via* several significant signaling pathways including Wnt, PI3K-Akt, FoxO, Apoptosis, and Metabolic signaling pathways. In addition, we discovered a novel lncRNA and named it lnc-GAM, then we demonstrated that lnc-GAM could regulate steroidogenesis and cell proliferation in POGCs. These results provide valuable resources for studying lncRNA and mRNA involved in POGCs apoptosis and can help clarify the molecular mechanisms of ovarian atresia and granulosa cell apoptosis.

## Data availability statement

The datasets presented in this study can be found in online repositories. The names of the repository/repositories and accession number(s) can found in NCBI BioProject PRJNA854769/Supplementary material.

## Ethics statement

The animal study was reviewed and approved by the Ethics Committee of the Anhui Agricultural University under permit no. AHAU20201025.

## Author contributions

HZ and YL: planned and designed the experiments. ZH and QX: methodology. NZ and JW: investigation. XZhe: data curation. YD: formal analysis. HZ: writing—original draft. XZha: writing—review and editing. ZY: supervision. ZY and XZha: funding acquisition. All authors have read and agreed to the published version of the manuscript.
