# Supplementary figures and images for: Integrated analysis of lncRNA and mRNA for the apoptosis of porcine ovarian granulosa cells after polyphenol resveratrol treatment
Source: Front Vet Sci. 2023 Jan 10;9:1065001. doi: 10.3389/fvets.2022.1065001 (PMC9872129; doi:10.3389/fvets.2022.1065001)

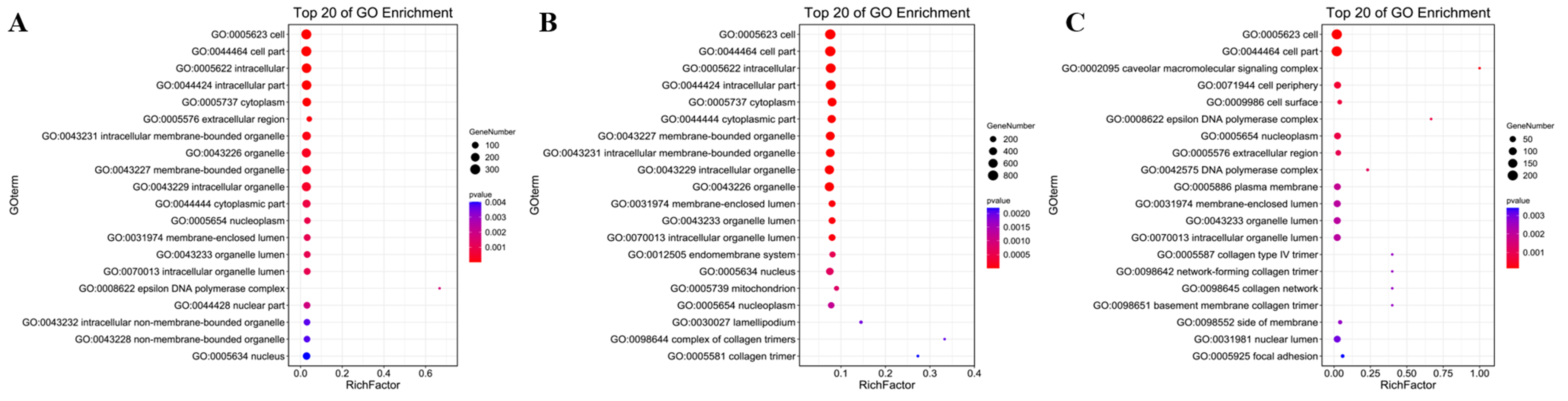

Supplement: Supplementary file 9 [file Image_1.TIF]
